# Body condition score and weight are effective targeted selective treatment indicators for gastrointestinal nematodes in premating ewes

**DOI:** 10.1002/vetr.5923

**Published:** 2025-12-02

**Authors:** Eiry G. Williams, Peter M. Brophy, Hefin W. Williams, Serian R. Evans, Heather McCalman, Rhys A. Jones

**Affiliations:** ^1^ Department of Life Science Aberystwyth University Aberystwyth UK; ^2^ Hybu Cig Cymru Aberystwyth UK

**Keywords:** anthelmintic resistance, gastrointestinal nematodes, sheep reproduction, targeted selective treatment

## Abstract

**Background:**

Ewe reproductive performance is key for flock profitability but can be impacted upon by gastrointestinal nematode (GIN) infections. Farmers commonly deworm ewes pre‐mating, yet concerns regarding anthelmintic resistance increase pressure to reduce wormer use.

**Methods:**

This study retrospectively assessed pre‐mating targeted selective treatment (TST) indicators in a flock of 354 ewes split into anthelmintic treatment and control groups. Conway–Maxwell–Poisson and binomial regression analyses were used to identify TST indicators associated with reproductive performance.

**Results:**

There was no significant difference in overall scanned litter size between treated and untreated ewes. However, ewes with a body condition score (BCS) less than 3 or a weight less than 60 kg that were not dewormed had significantly lower litter sizes (mean 1.2 and 1.41, respectively) than those treated (1.94 and 1.8, respectively) or with higher condition/weight regardless of worming status (*p* < 0.05).

**Limitations:**

Parasite diagnostics were not undertaken on all individual ewes, and the study assumes that differences identified in the reproductive performance of treated and untreated groups were due to GIN infection.

**Conclusion:**

These findings suggest that BCS and weight are effective indicators for implementing TST pre‐mating, enabling reduced anthelmintic use without compromising reproductive outcomes.

## INTRODUCTION

Ewe reproductive performance is among the greatest determinants on flock productivity and profitability.^1^, ^2^ Reproductive performance can be measured by the reproduction rate, which can be defined as the number of lambs produced, either at mid‐pregnancy, at birth or weaning, on average by each ewe within a flock. Farmers place great emphasis on optimally managing ewes in the weeks leading up to and during mating. For example, ewe ovulation rates and embryo survival, two of the main drivers for optimal reproductive rates, can be positively influenced by active management during this period,^3^ which may include the provision of increased nutrients and more focus on controlling ewe condition and disease.

Among these diseases, parasitic gastroenteritis (PGE) caused by gastrointestinal nematode (GIN) infections is often regarded as the most challenging to control in sheep flocks. GIN infections are among the most detrimental pathogens to sheep health and welfare, with an estimated €120–477 million annual cost to the European sheep meat industry.^4^, ^5^ Since GINs suppress their host's appetite and deplete ewe nutrient attainment, their subsequent impact on ewe reproductive performance could be substantial especially as undernourished ewes have lower ovulation and embryo survival rates.^6^ Experiments have demonstrated that GIN infections can negatively influence ewe reproductive performance,^7^, ^8^ albeit these impacts are more commonly observed in ewe lambs.^9^, ^10^ For ewes, which are likely to display stronger adaptive immunity against all common pathogenic GIN species in temperate regions, questions remain regarding the impact of GIN on reproductive performance during the mating period and whether anthelmintic treatment is warranted.

Despite this uncertainty and the lack of scientific studies focusing on GIN control in ewes pre‐mating, commonly shared advice, including that published by the SCOPS group in the UK, advise that treatment of ewes pre‐mating is unwarranted in most instances and such unnecessary treatment can increase anthelmintic resistance development in GIN populations.^11^ However, deworming ewes pre‐mating is commonly undertaken practice with between 42% and 63% of British and Irish farmers deworming their ewes against GIN pre‐mating.^12^, ^13^ This high rate implies that there are ongoing concerns on farms that reproductive performance of ewes is perceived to be influenced by GIN infections. However, as faecal egg count (FEC) monitoring is not regularly undertaken on ewes on British farms,^13^ it is feasible that these concerns are unfounded and treatment at this stage is simply driven by routine treatment strategies devised in the era before anthelmintic resistance mechanisms were fully understood. If so, major gains in reducing anthelmintic use on sheep farms may be achieved if deworming practices can be altered at mating, a reduction that can aid the slowing of anthelmintic resistance development in GIN populations.

One such alteration to deworming strategies may be the application of targeted selective treatment (TST), where treatment decisions are made at the individual animal rather than flock level. The benefits of TST are well established in lambs, with studies consistently showing that deworming individual lambs based on growth rate parameters can lead to significant decreases in dewormer use while having no detrimental impact on lamb performance.^14^, ^15^, ^16^, ^17^ However, limited studies exist in relation to TST of ewes. Those studies have demonstrated that only ewes below a certain body condition score (BCS) threshold may benefit from treatment.^18^ For example, only ewes with a BCS less than 2.75 prior to mating improved conception rates after deworming according to Calvete et al.,^19^ while ewe FECs are known to be unevenly distributed within a flock, with higher FECs observed in low BCS ewes.^20^ These studies indicate that ewe BCS may be the optimal TST determinant in ewes; however, BCS is subjective and cannot be autonomously recorded, and as such alternative TST determinants may be more practically implemented if deemed appropriate.

The aim of this study was to assess the feasibility of TST of ewes pre‐mating to maximise ewe reproductive performance while minimising anthelmintic administration. Furthermore, the study aimed to retrospectively assess various TST indicators for their suitability to guide TST against GIN at this point.

## MATERIALS AND METHODS

### Study design and data collection

Ethical approval was received for this work by the Aberystwyth University Animal Welfare and Ethical Review Board on 10 August 2020. This study was conducted on a commercial sheep farm in Ceredigion, Wales, UK. A proportion of the flock, totalling 354 Welsh Mule ewes were allocated randomly into two groups: anthelmintic treatment (*n* = 182) and control (*n* = 172), with groupings recorded via ewe electronic identification. Ewes were flushed, where they were given access to an increased plane of nutrition, over a 3‐week period before mating alongside a teaser ram. Ewe age ranged from yearlings (18 months of age at mating) to 8 years old. Ewes in the anthelmintic treatment group were administered with 0.2 mg/kg bodyweight of ivermectin (Animec, Chanelle Pharma) orally, pre‐mating. Ewe BCS,^21^ weight and dag score^22^ were recorded for all ewes pre‐flushing (3 weeks before mating), pre‐mating (1 day before ram introduced) and post‐mating (11 weeks after mating commenced). The same individual assessed ewes on each occasion to maximise consistency. A total of six rams were introduced to these ewes. All ewes were scanned using ultrasound 70‒90 days into gestation to determine pregnancy status and litter size.

A group FEC was performed pre‐mating where a composite sample of 10 individual 3 g faecal samples from the whole flock^23^ was analysed using the mini flotac method, which has a sensitivity of 5 eggs per gram (EPG).^24^ This composite faecal sample was also subjected to nematode culture^25^ and nemabiome analysis.^20^ The pasture that the sheep were grazing was assessed weekly, with sward height measured using a plate meter to calculate sward density (kg/DM per ha) and quality following Farming Connect guidelines.^26^

The energy utilisation (EU) of each ewe during the pre‐mating period was calculated in this study following the methodology of Greer et al.^14^ adapted to include ewe‐specific parameters identified following a literature search. Ewe‐specific values for calculating metabolisable energy for maintenance requirements (0.491 × liveweight^0.75^)^27^ and net energy for 1 kg liveweight gain (26 MJ)^28^ were identified during this search and were used in an updated model. All other formulas used in the calculation were those used by Greer et al.^14^ to calculate lamb EU as no ewe‐specific formulas or values could be found for these parameters.

### Statistical analysis

All statistical analysis was conducted in R using the glmmTMB and DHARMa packages.^29^, ^30^ Regression analysis was conducted to identify factors associated with ewe reproductive performance using measures relating to scanned litter size. The analysis also aimed to identify if anthelmintic treatment of ewes exhibiting specific physical or physiological traits, which may be indicative of a higher GIN burden would be beneficial in enhancing reproductive performance. Two measures of reproductive performance were analysed, scanned litter size and multiple lamb pregnancy status (i.e., carrying twins or triplets, rather than single lambs or being barren). Although Poisson or negative binomial regressions are regarded as the standard tools for analysing the relationship between count‐dependent variables and independent variables, significant under dispersion was detected within the scanned litter size count data, which undermined the assumption of Poisson and negative binomial regressions. Subsequently, a Conway–Maxwell–Poisson (CMP) regression was performed as this regression is regarded as a viable alternative for analysing under‐ or over‐dispersed count data.^31^ No significant under or over dispersion was detected in any CMP regression model performed during the analysis of the data set (Figure S1), which indicated that the CMP model was appropriate.^29^ A binomial regression was implemented to undertake analysis of ewe multiple lamb pregnancy status.

Factors assessed for their association with reproductive performance included physical or physiological traits considered to be indicative of a higher GIN burden. These were assessed for their suitability to inform GIN TST strategies to maximise reproductive performance in ewes by analysing their association with reproductive performance while interacting with the impact of anthelmintic treatment (i.e., dewormed or not dewormed). These traits included ewe BCS pre‐flushing and pre‐mating, weight pre‐flushing and pre‐mating, EU, age, weight loss or BCS loss during flushing and dag score. For each of these traits, ewes were retrospectively split into two groups for the purpose of the analysis. For BCS, thin ewes were classified as those with a BCS below 3, while for weight, light ewes were classified as those weighing less than 60 kg. For EU, ewes were split into two groups: high EU or low EU. The threshold for determining which category ewes were assigned to (0.7) was based on the average of two EU thresholds that have been identified as the optimal for TST of lambs based on this methodology in the literature, 0.66^14^ and 0.74.^32^ For dag score, ewes were classified as having dags present or not present pre‐mating, while for BCS loss and weight loss, ewes were classified as either losing or not losing BCS and weight, respectively. As yearling ewes are regarded as being more vulnerable to GIN,^33^ ewes were also classified via their age based on a threshold of less than 2 years of age (yearlings) or older than 2 years of age (ewes). This analysis led to an evaluation of whether treating ewes exhibiting physical or physiological traits considered to be indicative of a higher GIN would be significantly beneficial regarding their reproductive performance.

Figures presenting the results of both the CMP and binomial regression analyses were created using the ggeffects package and ggpredict function in R studio.^34^

## RESULTS

### Descriptive results

A descriptive summary of the cohort of ewes used in this study is presented in Table 1.

**TABLE 1:** Descriptive statistics regarding ewes used in this study.

Variable	Mean	SD	Category	*N*	Percent of ewes
Age	3.6	1.3	Yearlings	91	25.6
			Ewes	263	74.4
BCS pre‐flushing	3.28	0.39	<3	41	11.6
			≥3	313	88.4
BCS pre‐mating	3.38	0.34	<3	22	6.2
			≥3	332	93.8
Weight pre‐flushing (kg)	64.33	6.68	<60	90	25.4
			≥60	264	74.6
Weight pre‐mating (kg)	67.38	7.00	<60	47	13.3
			≥60	307	86.7
Scanned litter size	1.81	0.54	0	6	1.7
			1	74	20.9
			2	255	72
			3	19	5.4

Abbreviation: BCS, body condition score.

A group strongyle of 215 EPG was recorded pre‐treatment. *Trichostrongylus* spp. (35%) was the most abundant GIN species infecting the flock, followed by *Cooperia curticei* (22%) and *Teladorasgia cicumcintca* (18%), according to nemabiome analysis. No non‐strongyle GIN species were detected during FEC analysis.

### Ewe litter size in relation to targeted selective treatment indicators

**FIGURE 1:**
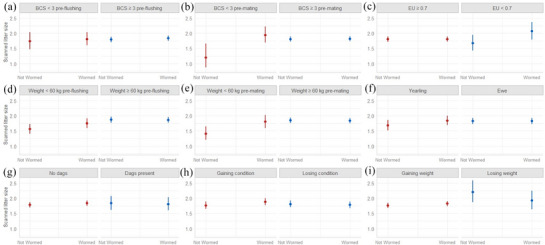
Visualisation of Conway–Maxwell–Poisson regression analysis of the reproductive performance as measured by scanned litter size of ewes retrospectively categorised based on their worming status and targeted selective treatment indicator grouping. Error bars represent 95% confidence intervals. A significant effect of deworming ewes on scanned litter size was observed in three multivariable models where ewe body condition score (BCS) pre‐mating (b) (*p* = 0.007), energy utilisation (EU) (c) (*p* = 0.043) and ewe weight pre‐mating (e) (*p* = 0.015) were included as fixed factors, respectively. Significant interaction effects between deworming status and BCS pre‐mating (b) was observed where the reproductive performance of dewormed ewes was significantly higher than non‐dewormed ewes with a BCS below 3 only (*p* = 0.009). Significant interaction effects between deworming status and weight pre‐mating (e) was also observed where the reproductive performance of dewormed ewes was significantly higher than non‐dewormed ewes with a weight below 60 kg only (*p* = 0.018). There was no significant effect of deworming ewes when categorised by their pre flushing BCS (a), weight pre flushing (d), age (f), dag score (g), condition gain status (h) or weight gain status (i) on scanned litter size (p > 0.05).

Figure 1 shows results of CMP regression analysis comparing reproductive performance of ewes categorised by certain physical or physiological traits that were either dewormed or not dewormed. There was no significant difference in the scanned litter size of dewormed ewes (mean =  1.83, 95% confidence interval [CI] = 1.75‒1.91) and ewes that were not dewormed (mean = 1.79, 95% CI = 1.71‒1.88) pre‐mating (*p* = 0.516). However, a significant effect of deworming ewes on scanned litter size was observed in multivariable models where ewe BCS pre‐mating (*p* = 0.007), ewe weight pre‐mating (*p* = 0.015) and EU (*p* = 0.043) were included as fixed factors. Significant interaction effects between deworming and either BCS pre‐mating (*p* = 0.009) or ewe weight pre‐mating (*p* = 0.018) were also seen in respective models. Ewes with a BCS less than 3 that were not dewormed had a mean scanned litter size of 1.20 (95% CI = 0.87–1.66), which was significantly lower compared to ewes with a BCS less than 3 that were dewormed (1.94, 95% CI = 1.69–2.23) and ewes with a BCS 3 or more that were either dewormed (1.82, 95% CI = 1.73–1.91) or not dewormed (1.81, 95% CI = 1.73–1.90) (*p* < 0.05). Ewes with a weight less than 60 kg pre‐mating that were not dewormed had a mean scanned litter size of 1.41 (95% CI = 1.21–1.65), which was significantly lower compared to ewes with a weight less than 60 kg that were dewormed (1.80, 95% CI = 1.59–2.03) and ewes with a weight 60 kg or more that were either dewormed (1.83, 95% CI = 1.75–1.92) or not dewormed (1.85, 95% CI = 1.76–1.94) (*p* < 0.05). There was no significant effect of deworming or any significant interaction effect between deworming and any other fixed factor in the other multivariable models created (Figure 1; *p* > 0.05).

### Proportion of ewes carrying multiples in relation to targeted selective treatment indicators

**FIGURE 2:**
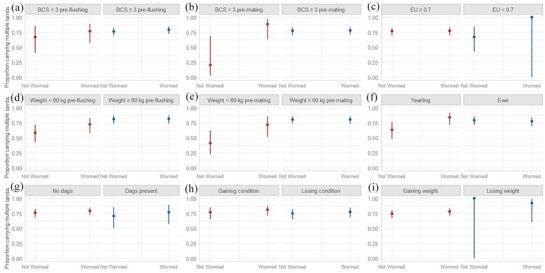
Visualisation of binomial regression analysis of the reproductive performance as measured by the proportion of ewes carrying multiple lambs retrospectively categorised based on their worming status and targeted selective treatment (TST) indicator grouping. Error bars represent 95% confidence intervals. A significant effect of deworming ewes on multiple lamb carrying status was observed in two multivariable models where ewe body condition score (BCS) pre‐mating (b) (*p* = 0.012) and ewe weight pre‐mating (e) (*p* = 0.035) were included as fixed factors, respectively. Significant interaction effects between deworming status and BCS pre‐mating (b) was observed where the reproductive performance of dewormed ewes was significantly higher than non‐dewormed ewes with a BCS below 3 only (*p* = 0.015). Significant interaction effects between deworming status and weight pre‐mating (e) was observed where the reproductive performance of dewormed ewes was higher than non‐dewormed ewes with a BCS below 3 only (*p* = 0.051). A significant interaction effects between deworming status and ewe age (f) was also observed where the reproductive performance of dewormed ewes was significantly higher than non‐dewormed yearling ewes only (*p* = 0.035). There was no significant effect of deworming ewes when categorised by their pre flushing BCS (a), energy utilisation (c), weight pre flushing (d), dag score (d), condition gain status (h) or weight gain status (i) on the proportion carrying multiple lambs (p > 0.05).

Figure 2 shows the results of binomial regression analysis comparing reproductive performance of ewes categorised by certain physical or physiological traits that were either dewormed or not dewormed. There was no significant difference in the proportion of dewormed ewes carrying multiples (0.79, 95% CI = 0.73–0.84) compared to those not dewormed (0.76, 95% CI = 0.69–0.81) pre‐mating (*p* = 0.427). However, a significant effect of deworming ewes on the proportion of ewes carrying multiple lambs was observed in multivariable models where ewe BCS pre‐mating (*p* = 0.012) and ewe weight pre‐mating (*p* = 0.035) were included as fixed factors, respectively, while significant or near‐significant interaction effects between deworming and either BCS pre‐mating (*p* = 0.015), ewe weight pre‐mating (*p* = 0.051) or ewe age category (*p* = 0.035) were seen in respective models.

The proportion of ewes with a BCS less than 3 that were not dewormed carrying multiples was 0.2 (95% CI = 0.03–0.69), which was significantly lower compared to ewes with a BCS less than 3 that were dewormed (0.88, 95% CI = 0.63–0.97) and ewes with a BCS 3 or more that were either dewormed (0.78, 95% CI = 0.71–0.84) or not dewormed (0.77, 95% CI = 0.7–0.83) (*p* < 0.05). The proportion of ewes with a weight less than 60 kg and not dewormed carrying multiples was 0.41 (95% CI = 0.23–0.62), which was significantly lower compared to ewes with a weight less than 60 kg that were dewormed (0.72, 95% CI = 0.52–0.86) and ewes with a weight 60 kg or more that were either dewormed (0.80, 95% CI = 0.73‒0.86) or not dewormed (0.81, 95% CI = 0.74–0.86) (*p* < 0.05). The proportion of non‐dewormed yearlings carrying multiples was 0.63 (95% CI = 0.48–0.77), which was significantly lower compared to yearlings that were dewormed (0.84, 95% CI = 0.71–0.92) and ewes that were either dewormed (0.77, 95% CI = 0.69‒0.84) or not dewormed (0.79, 95% CI = 0.72–0.85) (*p* < 0.05). There was no significant effect of deworming or any significant interaction effect between deworming and any other fixed factor in the other multivariable models created (Figure 2).

## DISCUSSION

This study provides evidence that TST of ewes pre‐mating, using BCS or weight as a determinant, is a viable alternative to blanket treatment for farmers who seek to treat their ewes against GIN pre‐mating. Importantly, it was estimated that only a small proportion of ewes from this flock benefited from treatment to enhance reproductive performance. Specifically, applying TST to ewes below a BCS or weight threshold of 3 and 60 kg, respectively, immediately prior to mating, maximised ewe reproductive performance while anthelmintics would only be administered to either 6.2% or 13.3% of this flock depending on the indicator used. Blanket deworming of ewes pre‐mating is now widely discouraged due the effect it has on increasing anthelmintic resistance development, especially as ewes develop immunity against most GIN species. However, treating ewes at this time with an anthelmintic of proven efficacy may be beneficial to maximise reproductive performance in some instances,^7^ with a high proportion of farmers in Great Britain known to deworm their ewes pre‐mating.^13^ Our results suggest that pre‐mating treatment is very likely to be beneficial only to certain ewes, if any, thus making TST a feasible GIN control strategy in this instance.

The reproductive performance of ewes weighing less than 60 kg and those ewes with a BCS less than 3 pre‐mating was significantly improved if dewormed, while there was no benefit in deworming ewes weighing 60 kg or more or with a BCS of 3 or higher. It is now widely accepted in the industry that an ewe's BCS is among the strongest predictor of ewe reproductive performance with benchmarks for ewe BCS pre‐mating of 3‒3.5 widely targeted for flocks.^35^ The importance of BCS for optimal reproduction performance is widely acknowledged, with ewes carrying twins tending to have a higher BCS on average at mating compared to ewes carrying a single lamb.^36^ Additionally, barren ewes are likely to have had a lower BCS on average at mating than those in lamb.^36^ Several previous researchers have investigated the relationship between BCS and GIN burden in both sheep and goats. These studies consistently show that there is a negative correlation between BCS and FEC, with ewes having a lower BCS being more likely to suffer from higher worm burdens.^20^, ^37^, ^38^ Considering the negative implications of poor BCS on reproductive performance and the fact that GIN infection burden may be a contributor to low BCS, BCS can be used as an effective TST indicator for ewes pre‐mating. The use of BCS as a TST indicator in ewes was previously investigated by Calvete et al.^19^ who found that deworming ewes with a BCS less than 2.75 pre‐mating increased fertility and improved conception rates. Similarly, Cornelius et al.^18^ found that periparturient ewes with a lower BCS showed greater relative BCS response to anthelmintic treatment compared to ewes with a higher BCS.

As weight is strongly correlated with BCS^39^ and is also known to be a key predictor of ewe reproductive performance,^36^ it is likely that similar reasons can explain why only ewes weighing less than 60 kg benefited from deworming pre‐mating. It is therefore likely that both BCS and weight would be practical measures for farmers to base TST of ewes pre‐mating on. In our study, 6.2% of ewes had a BCS less than 3, while 13.2% weighed less than 60 kg. This would suggest that TST based on BCS could lead to lower deworming rates while reproduction rates are maximised. However, BCS is a subjective measure, and cannot be automated at present for sheep, although recent automated BCS recorders are now available for cattle.^40^, ^41^ Automatic weighing systems are currently available; thus, TST of ewes based on weight could be an efficient practice on farms with such systems. However, although this study identified 60 kg as an optimal threshold for TST in Mule ewes, it is unlikely that this threshold would be appropriate for other breeds, which may have larger or smaller frames. Furthermore, it is unlikely that an optimal weight threshold for TST of ewes can be obtained in flocks of mixed breeds and/or with varying frame size. To alleviate this issue, growth models could be employed where the optimal weight for each individual ewe could be calculated based on factors such as reference weight, breed, age, season and pasture availability. Despite the suggestion that such models may be impractical on a commercial scale in any case,^42^ it has been demonstrated that an EU model, which predicts expected lamb weights, can successfully guide TST in lambs.^14^ Nevertheless, our study failed to demonstrate that this EU model, when adapted for ewes using the limited data available in the literature could effectively guide TST in ewes pre‐mating.

Despite demonstrating the suitability of using ewe BCS or weight post‐flushing as TST indicators, albeit in a flock where the vast majority of ewes were not thin, the previous BCS and weight of ewes measured pre‐flushing were not shown to be suitable TST indicators. This suggests that the timing of assessing ewe BCS or weight for TST is vital for enabling optimal selection of ewes requiring treatment. In our study, it is likely that ewe BCS/weight at the end of the flushing period was a better reflection of those ewes suffering from PGE and thus was a better determinant of ewes that would benefit from being dewormed. For these ewes in a poor condition post‐flushing, even access to an increased plane of nutrition failed to improve or maintain appropriate condition potentially due to GINs suppressing appetite and impacting upon digestive efficiency.^6^ Ewes in a poorer condition pre‐flushing may have been so due to historic nutritional deficiencies or health issues, which were no longer present; thus, the increased plane of nutrition attained during the flushing period allowed target BCS to be achieved before mating commenced. Nevertheless, no direct parasitological measurements were made on individual animals in this study; thus, further studies are needed to confirm this hypothesis.

Our study also demonstrated that age may have potential to be used as a TST indicator as it was shown that deworming yearlings significantly increased the proportion of those ewes that carried multiples, while the same effect was not observed for older ewes. Nevertheless, the degree of difference in the reproduction performance of dewormed and non‐dewormed yearling ewes was not as substantial as the differences observed between dewormed and non‐dewormed thin (BCS < 3) or light (<60 kg) ewes. Age was also not shown to be a suitable TST indicator when considering litter size as the reproductive performance indicator. However, no ewe lambs were present in this study, and further investigation would be required to identify optimal TST determinants for this age class, which are likely to be more susceptible to GIN.

There was no benefit seen in deworming ewes displaying dag scores on their reproductive performance. Farmers who dewormed ewes based on dag score were found to treat their ewes significantly more often.^13^ Considering that a wide range of issues can lead to sheep scouring, many of which may not be the result of parasitic infection, dag presence or scores may not be as appropriate a TST indicator compared to other measures. One common aggravator of scouring is the grazing of lush pastures,^43^ a common occurrence pre‐mating as ewes are flushed in an attempt to improve condition and subsequent reproductive performance. Subsequently, reliance on dag score as TST indicator pre‐mating could lead to the unnecessary treatment of ewes.

Although this study clearly demonstrates the feasibility of using ewe BCS and/or weights as indicators for TST against GIN pre‐mating, significant barriers exist as to the general uptake of TST in sheep flocks. These barriers include a concern that deliberately withholding treatments for certain sheep may negatively affect flock productivity.^44^ While effective communication of the benefit of TST in reducing the rate of anthelmintic resistance development can alleviate these concerns,^44^ the need to control other parasites during this period may also make TST unviable. For example, in the UK and Ireland, both fasciolosis and sheep scab are prominent diseases of sheep traditionally seen from the autumn mating period onwards. As well as treating GIN, broad‐spectrum macrocyclic lactones and certain benzimidazoles also treat sheep scab and fasciolosis, respectively, while combination products that contain multiple narrow‐spectrum drugs to treat GIN and liver fluke infections are also commonly administered.^45^ Broad‐spectrum or combination treatments against scab, fasciolosis or other parasitic disease may therefore be administered without necessarily having an aim or a demonstrated need to control GIN at that specific time.^46^ Williams et al.^13^ found that British farmers using combination products against fluke and GIN and those who routinely use macrocyclic lactone products to prevent sheep scab were significantly more likely to deworm ewes pre‐mating, which highlights the key influence such practices have on likelihood of over deworming ewes. As TST is not a viable strategy for controlling liver fluke or sheep scab at present, use of narrow‐spectrum treatments should be encouraged to enable the optimal targeting of problematic parasite species at flock and individual animal level.^45^ Simultaneously, there is also a need to accelerate the uptake of diagnostic testing to ensure that specific parasite species infections are correctly identified and treated with an appropriate product at the right time, ideally with narrow‐spectrum products. In regard to GINs, use of FEC has been associated with reduced likeliness to deworm ewes pre‐mating.^13^ Uptake of FEC is widely encouraged,^11^ albeit with disappointing uptake rates.^13^ Nevertheless, this study does demonstrate one issue of strictly using FEC to guide whole flock targeted treatment, which is that in instances where flock FEC is low and a subsequent decision is made not to deworm, the performance of a small proportion of individual animals within the flock requiring treatment may be impaired. There are also challenges surrounding TST as discussed, which may also include difficulties in determining optimal treatment thresholds, which may similarly lead to animals requiring treatment not being dewormed,^17^ an issue that could present itself when applying the TST thresholds identified in this study on other farms. This issue may be particularly pronounced on farms where a larger proportion of ewes fall below the TST thresholds identified in this study, not solely due to GIN infection but also as a result of other parasitic or non‐parasitic diseases, suboptimal nutrition or poor management practices, all of which can contribute to reduced BCS or weight. Further investigation into factors that influence variability in TST thresholds between flocks and over time is therefore necessary, as is research to identify measures that may be incorporated into TST decision‐making systems to account for such factors. Interestingly, incorporating group FEC has been shown to reduce between farm variability in models predicting individual ewe FEC, and as such, FEC data may play an integral role in future TST decision systems.^20^

## CONCLUSION

The reproductive performance of only Welsh Mule ewes with a BCS less than 3 or which weighed less than 60 kg pre‐mating benefited significantly from anthelmintic treatment. Therefore, TST based on ewe pre‐mating BCS or weight may maximise flock performance while reducing anthelmintic use, and subsequently, anthelmintic resistance development. This study provides novel evidence of potential TST markers pre‐mating that should be further confirmed in studies including direct parasite diagnoses of evaluated animals. Considering that this study was based on a single flock, further research is also required to identify optimum BCS and weight thresholds for TST of other breeds and production systems and to investigate the feasibility of incorporating flock level variables, potentially FEC data, to aid the determination of such thresholds.

## AUTHOR CONTRIBUTIONS

*Conceptualisation, methodology, investigation, formal analysis and writing—original draft*: Eiry Williams. *Writing—review and editing and supervision*: Peter Brophy and Hefin Williams. *Writing—review and editing*: Serian Evans and Heather McCalman. *Conceptualisation, methodology, formal analysis, funding acquisition, writing—review and editing and supervision*: Rhys Jones.

## CONFLICT OF INTEREST STATEMENT

This study was administered under the KESS2 scheme and part funded by Hybu Cig Cymru/Meat Promotion Wales. KESS2 is part funded by the European Social Fund through the European Union's Convergence Programme (West Wales and the Valleys) administered by the Welsh Government.

## ETHICS STATEMENT

Ethical approval was received for this work by the Aberystwyth University Animal Welfare and Ethical Review Board on 10 August 2020. This study was conducted on a commercial sheep farm in Ceredigion, Wales, UK.

## Supporting information



Supporting information

## Data Availability

The data that support the findings of this study are available from the corresponding author upon reasonable request.
