# Supplementary material for: Body condition score and weight are effective targeted selective treatment indicators for gastrointestinal nematodes in premating ewes
Source: Vet Rec. 2025 Dec 2;198(3):e85–91. doi: 10.1002/vetr.5923 (PMC12857528; doi:10.1002/vetr.5923)
Supplement: Supplementary file 1 — Supporting information [file VETR-198--s001.pdf]

Supplementary Figures

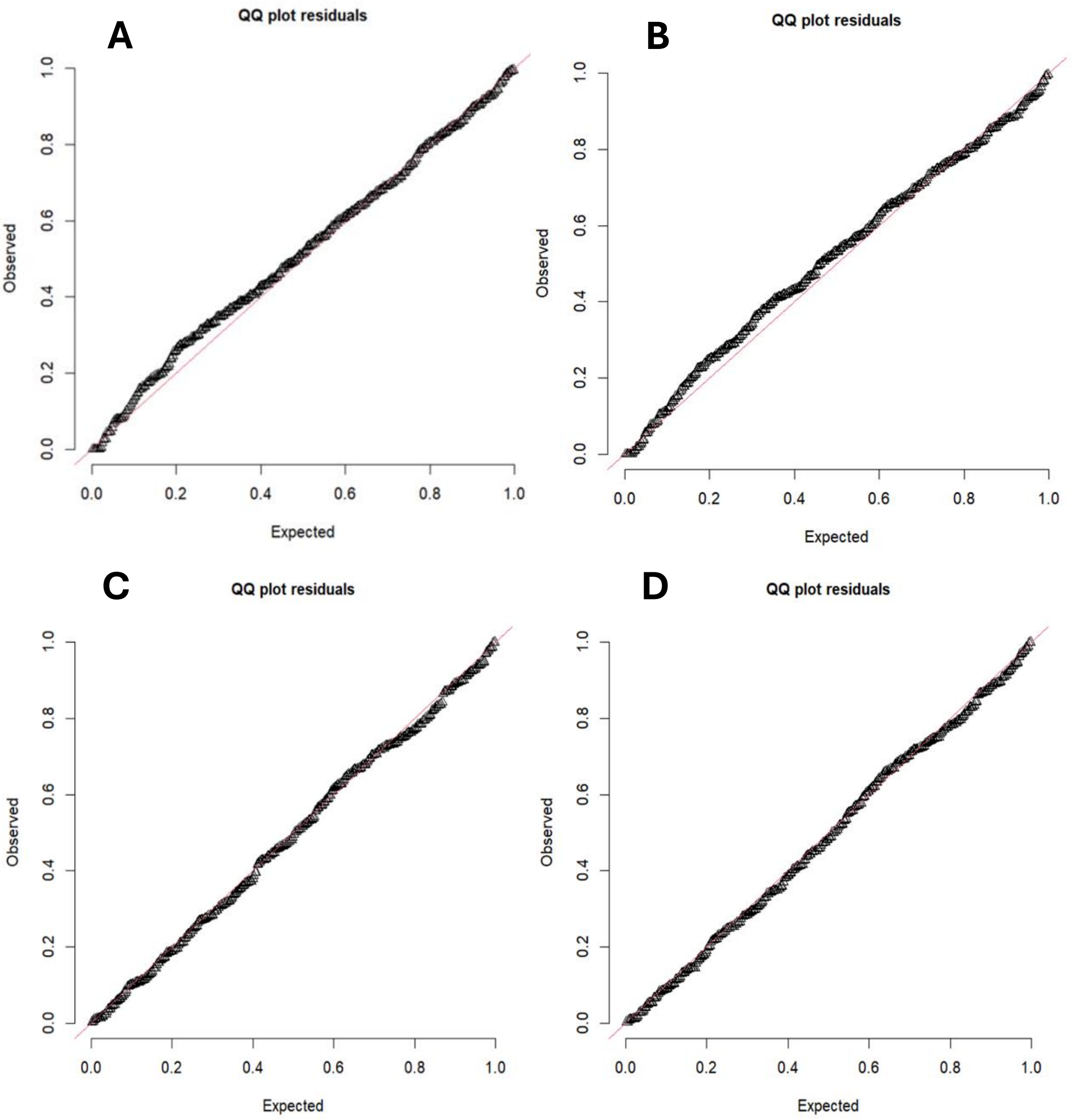

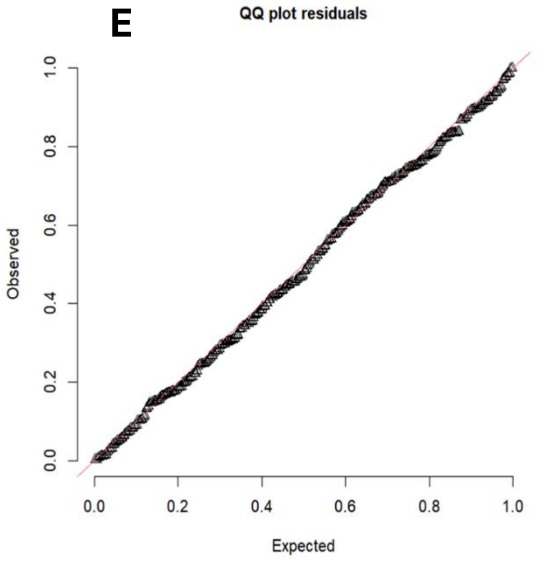

Figure S1: QQ plots of model observed versus expected residuals. (A) Scanned litter size ~ Ewe BCS pre-mating category * Deworming status; (B) Scanned litter size ~ Ewe weight pre-mating category * Deworming status; (C) Ewe multiple lamb status ~ Ewe BCS pre-mating category * Deworming status; (D) Ewe multiple lamb status ~ Ewe weight pre-mating category * Deworming status; (E) Ewe multiple lamb status ~ Ewe age category * Deworming status
